# Intranuclear Inclusions in Renal Tubular Epithelium in Immunodeficient Mice Stain with Antibodies for Bovine Papillomavirus Type 1 L1 Protein

**DOI:** 10.3390/vetsci2020084

**Published:** 2015-06-11

**Authors:** Elizabeth McInnes, Mark Bennett, Mandy O’Hara, Lorna Rasmussen, Peony Fung, Philip Nicholls, Michael Slaven, Robert Stevenson

**Affiliations:** 1 Cerberus Sciences, Unit 3, 49 Holland Street, Thebarton, SA 5031, Australia; E-Mails: lorna@cerberus.net.au (L.R.); peony@cerberus.net.au (P.F.); bob@cerberus.net.au (R.S.); 2 School of Veterinary and Life Sciences, Murdoch University, South Street, Murdoch, WA 6150, Australia; E-Mails: m.bennett79@ymail.com (M.B.); a.ohara@murdoch.edu.au (M.O.); P.Nicholls@murdoch.edu.au (P.N.); m.slaven@murdoch.edu.au (M.S.)

**Keywords:** mouse, intranuclear inclusions, renal tubular epithelium, bovine papillomavirus type 1 L1 protein

## Abstract

The kidneys from six immunodeficient mice examined by Cerberus Sciences and the Animal Resources Centre, displayed karyomegaly with pale eosinophilic, intranuclear inclusions upon histopathological examination. Electron microscopy performed on kidney tissue from 5/6 mice demonstrated margination of the chromatin in large nuclei. Laboratory tests were used to detect nucleic acid of papillomaviruses, polyomaviruses, circoviruses and anelloviruses (4/6 mice), a specific PCR was used to detect murine polyomavirus (1/6), and a panel of serological tests was used to detect seroconversion to major murine pathogens (1/6). All molecular and serological tests were negative. Immunohistochemistry using polyclonal anti-bovine papillomavirus type 1 (BPV-1) L1 antibody, Camvir monoclonal anti-papillomavirus antibody (directed against the seven amino acids GFGAMDF found in human papillomavirus (HPV) 16 L1 protein), a commercially available mixture of two monoclonal antibodies, anti-BPV-1 L1/1H8 + Camvir antibodies, and a monoclonal anti-Hsc70 antibody revealed specific, positive staining of murine renal tubular epithelial intranuclear inclusions in 6/6 mice using the anti-BPV-1 L1 containing antibodies only. Methyl pyronin green, PAS and Feulgen histochemical reactions revealed that the intranuclear inclusions did not consist of RNA, DNA or carbohydrate. An immunohistochemical method now exists that can be used to confirm and evaluate suspected cases of murine inclusion body nephropathy.

## 1. Introduction 

Veterinary pathologists examining mouse kidneys have occasionally noted the presence of large, eosinophilic intranuclear inclusions and karyomegaly in the renal tubular epithelium of immunodeficient mice [1]. Such observations have been made in kidneys collected from mice submitted for routine colony health monitoring, which have not undergone any experimental intervention [2]. When visualised using light microscopy, these intranuclear inclusions are morphologically similar to those observed in certain DNA virus infections (e.g., adenovirus, cytomegalovirus, and polyomavirus) [3]. Previously performed ultrastructural studies have confirmed chromatin margination and the presence of electron lucent material within affected nuclei, however no viral particles or other pathogens have yet been identified [2]. Serology, microbiology, parasitology, necropsy, histopathology, PCR and immunohistochemistry investigations have, to date, all failed to detect a pathogen that might be associated with these murine renal intranuclear inclusion bodies, and therefore, the tubular inclusions have so far been considered an idiopathic, degenerative lesion [2]. 

Here, we report on investigations into the chemical nature of these murine renal tubular intranuclear inclusion bodies, and the serendipitous discovery of antibodies that bind to and specifically highlight them. 

## 2. Methods 

### 2.1. Animals and Necropsy

All details about the mice in this study are included in Table 1. All six mice originated from the Animal Resources Centre (ARC) in Perth, Western Australia. Two mice were submitted to Cerberus Sciences and four were necropsied by ARC staff, as part of routine health monitoring procedures. 

A complete necropsy was performed on all mice by a veterinary pathologist. The kidneys were examined macroscopically and samples of fresh kidney tissue from mice A, C, D, E and F were taken for molecular tests, with a small sub-sample fixed in glutaraldehyde for electron microscopy from these same mice. Kidneys were collected from all mice and fixed in formalin. The formalin-fixed kidney tissues were routinely processed and embedded in paraffin wax, sectioned at 3–4 μm and stained with hematoxylin and eosin, Feulgen reaction, methyl pyronin green and PAS reaction [4]. Light microscopy was performed on these tissues. No experimental procedures were performed on the animals in this study and all of the animals were housed and maintained in accordance with the Australian code for the care and use of animals for scientific purposes 8th edition (2013) [5]. 

**Table 1:** Information about the six mice analysed in this study including identification, breed, sex and age description.

Animal	Breed	Sex	Description	Necropsy
A	CBySmn.CB17-*Prkdc^scid^*/J	F	Ex-breeder	Cerberus
B	CBySmn.CB17-*Prkdc^scid^*/J	F	Young	Cerberus
C	NOD.CB17-*Prkdc^scid^*	F	Ex-breeder	ARC
D	NOD.CB17-*Prkdc^scid^*	M	Ex-breeder	ARC
E	NOD.CB17-*Prkdc^scid^*	F	Ex-breeder	ARC
F	B6.129S7-*Rag1^tm1M^°^m^*/J	F	Ex-breeder	ARC

### 2.2. Serology

A whole blood sample was collected by cardiocentesis from mouse B. The blood sample was allowed to clot for 30 minutes and was then centrifuged for five minutes at 604.8g and the serum was harvested and stored at −20 °C. Serum from mouse B was tested for antibodies against the following murine pathogens using the ELISA test: mouse hepatitis virus, mouse minute virus, mouse parvovirus, mouse norovirus, rotavirus, Theiler’s murine encephalomyelitis virus, pneumonia virus of mice, Sendai virus, *Mycoplasma pulmonis*, mouse cytomegalovirus, mouse adenovirus 1, reovirus type 3, lymphocytic choriomeningitis virus, ectromelia virus, mouse adenovirus 2, murine polyomavirus, *Clostridium piliforme*, hantavirus, *Encephalitozoon cuniculi*, cilia associated respiratory bacillus, K virus and mouse thymic virus. For the ELISA tests, the method of McInnes and co-workers (2011) [6] was used. 

### 2.3. Electron Microscopy

Kidney tissue in glutaraldehyde from mice A, C, D, E and F was postfixed in 1 % osmium tetroxides, dehydrated and embedded in resin blocks. Sections were stained en-bloc with 1% uranyl acetate and ultrathin sections were then stained with Reynold’s lead citrate and examined with a Hitachi H7500 (A) or CM 100 BioTwin (C & D) transmission electron microscope (Philips, Eindhoven, The Netherlands).

### 2.4. Polymerase Chain Reaction 

Total DNA was extracted from the kidneys of mice A, C, D, E, and F with commercially available kits (UltraClean^®^ Tissue & Cells DNA Isolation kit (MO BIO Laboratories, Inc, USA); DNEasy Tissue and Blood (Qiagen) according to the manufacturers’ instructions. For total DNA extracts from mouse A, a PCR reaction mixture contained the following components with final concentration of 20 mmol/L Tris-HCl (pH 8.5 at 25 °C), 50 mmol/L KCl, 2 mmol/L MgCl_2_, 200 µmol/L each dNTP (dATP, dCTP, dTTP and dGTP), 0.5 U of Taq polymerase (Fisher Biotec, Australia) and 0.2 µmol/L each primers specific for murine polyomavirus VP2 protein-coding region [7] in a final PCR reaction volume of 25 µL. The PCR was run on a Peltier thermal cycler (MJ Research, United States) with the following temperature program: an initial denaturing step at 94 °C for 5 min, then 40 cycles of 94 °C for 30 s, 60 °C for 30 s, 72 °C for 30 s. The PCR products were electrophoretically separated in 2% agarose E-gels (Invitrogen, Australia) and visualized with UV light [6].

A broad-spectrum papillomavirus PCR was performed on DNA extracts from mice C, D, E, and F, according to the method described in Forslund *et al*. (1999) [8]. Multiply primed rolling circle amplification was also performed on these same samples, following the method of Rector and co-authors (2004, 2005) [3,9].

### 2.5. Immunohistochemistry (IHC)

The antibodies used for the murine inclusion body IHC were: rabbit polyclonal anti-bovine papillomavirus L1 antibody (Dako, Australia) (dilution 1:300), Camvir monoclonal anti-papillomavirus antibody (directed against the seven amino acid linear epitope GFGAMDF found in HPV 16 L1 protein) [10] (dilution 1:300), mixed monoclonal anti-BPV-1 L1/1H8 + Camvir (Abcam) (dilution of 1:300) and rabbit monoclonal anti-Hsc70 antibody (EP1531Y) (Novus Biologicals, Australia) (dilution 1:400). 

Antigen retrieval was required for the latter three antibodies and was achieved by heating the sections in a Tris-EDTA pH 9 buffer using a microwave oven. Endogenous peroxidase activity was quenched using a 3% aqueous hydrogen peroxide solution. Prior to the application of the antibodies, all sections were pre-treated with 10% normal goat serum which was drained off without washing. The three papillomavirus antibodies required an incubation time of 30 minutes while the heat shock cognate antibody required 60 minutes. Negative control sections were treated with Tris buffered saline wash solution pH 7.8 and isotype control antibodies. Visualisation of all the reactions was achieved with Dako Envision+ Dual Link System-HRP (Dako, Australia) for 30 minutes followed by treatment with Dako Liquid DAB+ Substrate Chromogen System (Dako, Australia) for three minutes. The sections were then counterstained in Harris’s haematoxylin, dehydrated, cleared and cover-slipped.

## 3. Results 

### 3.1. Histopathology

**Figure 1:**
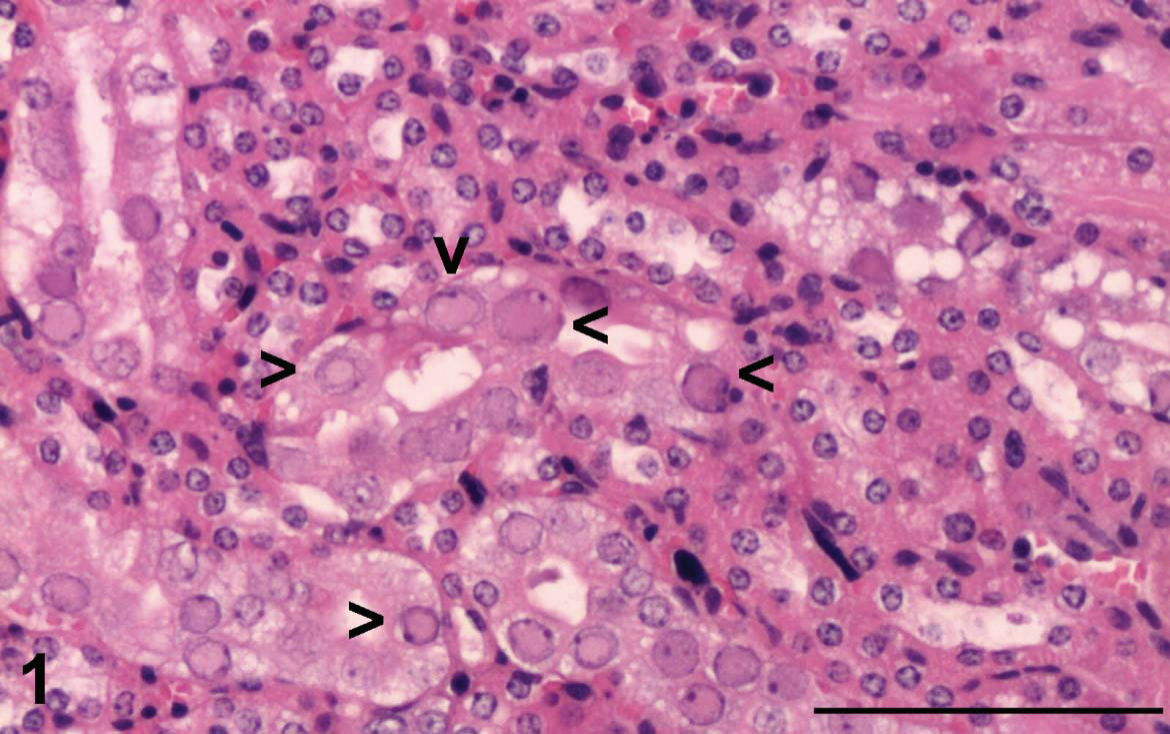
Renal cortical tubular intranuclear inclusion bodies (black arrowhead) from mouse F stained with haematoxylin and eosin. The black measurement bar represents 100 μm.

The kidneys from all six mice displayed mild to marked, focal to multifocal karyomegaly of tubular epithelial cells. Enlarged nuclei had marginated chromatin and contained pale, eosinophilic, inclusion bodies (Figure 1 and Figure 3a). The remaining cells in affected tubules contained abundant pale eosinophilic cytoplasm with occasional vacuolation. The majority of intranuclear inclusions were noted in tubular epithelium of the renal cortex, however, occasional inclusions were also noted in the tubular epithelium of the collecting ducts of the medulla. In addition, mouse A had a lymphoblastic lymphoma of the submandibular lymph nodes, spleen, liver, lungs and kidneys. Mouse D had concurrent moderate, subacute necrotizing pyelonephritis. 

### 3.2. Electron Microscopy

**Figure 2:**
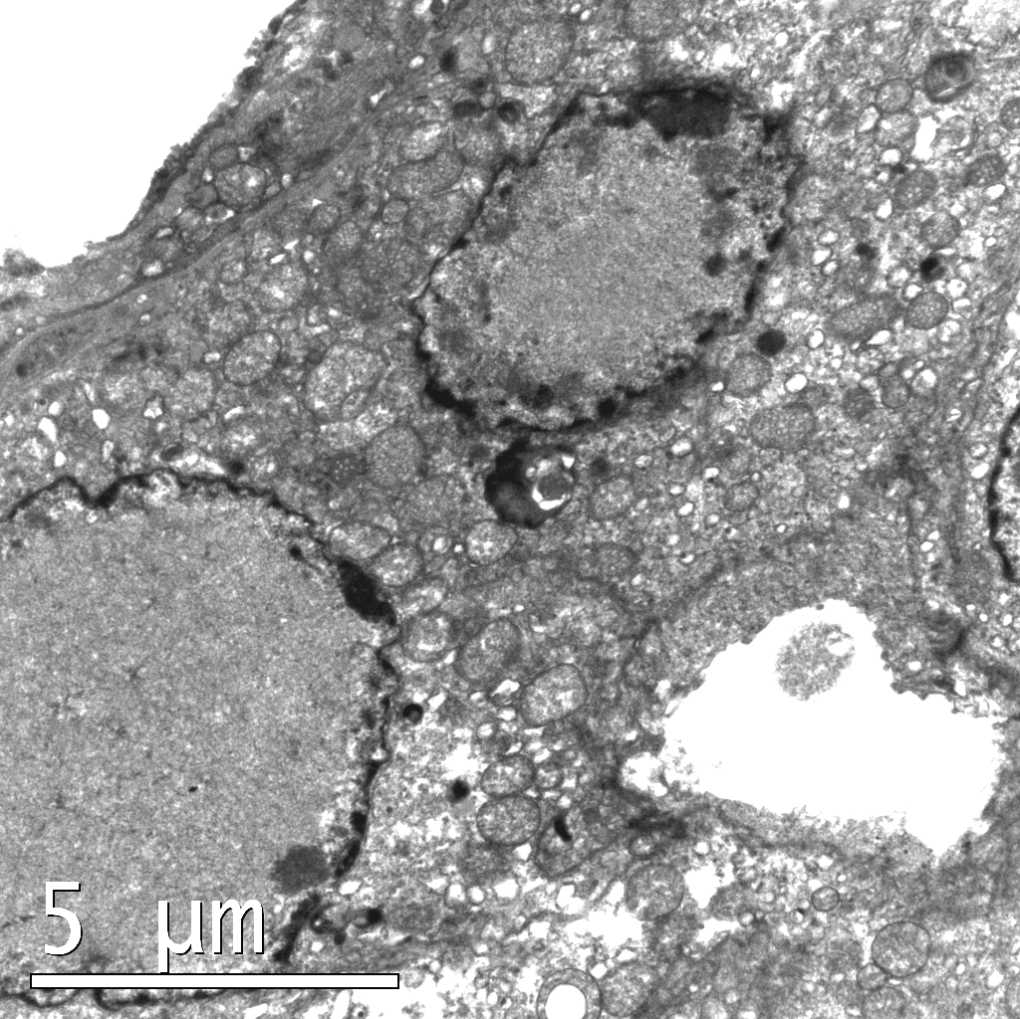
Chromatin displaced peripherally within affected nuclei, around abundant electron lucent material. Mouse A (lead citrate and uranyl acetate).

The renal tubular epithelium of the mice examined by electron microscopy displayed multiple, enlarged nuclei within tubular epithelial cells. Chromatin was displaced peripherally within affected nuclei, around abundant electron lucent material (Figure 2). No recognisable viral particles were observed within the enlarged tubular epithelial nuclei, however, at higher magnifications (up to × 100,000) the intranuclear material showed both a granular and fibrillar composition. 

### 3.3. Laboratory Tests

The kidneys from mouse A were negative for murine polyomavirus by PCR. Mice C, D, E and F were negative for papillomaviruses by PCR, and negative for small circular DNA genomes (*i.e.*, papillomaviruses, polyomaviruses, circoviruses, anelloviruses) by multiply primed rolling circle amplification. Serology for mouse B was negative for all organisms tested.

### 3.4. Histochemistry and Immunohistochemistry

**Figure 3:**
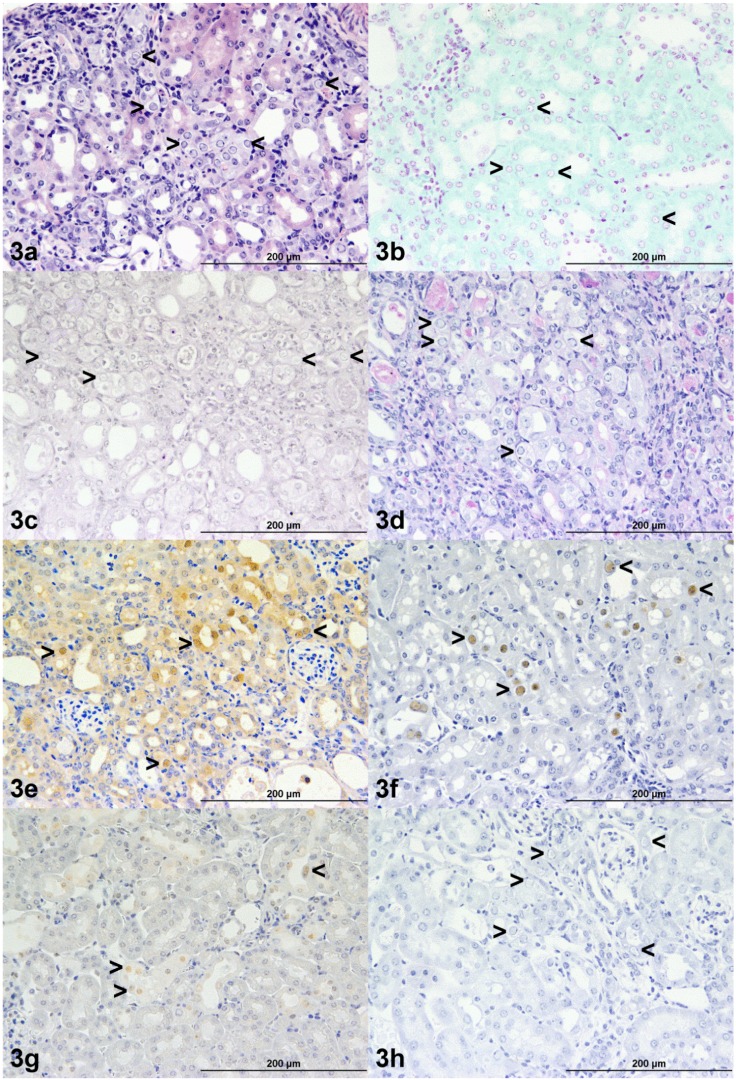
(**a**) Renal cortical tubular intranuclear inclusion bodies from mouse F stained with haematoxylin and eosin (**b**) Feulgen reaction, (**c**) Methyl Green Pyronin, (**d**) Periodic Acid Schiff’s reaction, (**e**) anti-Hsc70 monoclonal antibody, (**f**) polyclonal rabbit anti bovine papillomavirus type 1 L1 protein antibody, (**g**) a mixture of Camvir + monoclonal mouse anti bovine papillomavirus type 1 L1 protein antibody (the latter component’s epitope binding specificity is not known), (**h**) Camvir monoclonal antibody. The black arrows demonstrate the findings in the intranuclear inclusions.

Histochemical stains demonstrated that the inclusion bodies in all six mice did not consist of DNA, RNA or carbohydrate (Table 2, Figure 3b,c,d). Immunostaining with Hsc70 showed a mixture of cytoplasmic and nuclear staining in kidney cells, but this was not always related to the presence of the intranuclear inclusions (Table 2, Figure 3e). The anti-papillomavirus antibodies (polyclonal and monoclonal) that included reactivity against bovine papillomavirus type 1 L1 protein, specifically, consistently and strongly produced positive immunostaining of the intranuclear inclusions (Table 2, Figure 3f,g). In contrast, the Camvir monoclonal anti-papillomavirus antibody produced no immunostaining of murine kidney tissues (Table 2, Figure 3h). 

**Table 2:** The results of the histochemical and immunohistochemical staining used in this study.

Animal	A	B	C	D	E	F
H&E	+	+	+	+	+	+
Mixed monoclonal anti-BPV-1 L1/1H8 + Camvir	+	+	+	+	+	+
Rabbit polyclonal anti-bovine papillomavirus L1 antibody	+	+	+	+	+	+
Camvir monoclonal anti-papillomavirus antibody	–	–	–	–	–	–
Rabbit monoclonal anti-Hsc70	cyto > nuc	cyto > nuc	weak +	weak +	weak +	weak +
PAS	–	–	–	–	–	–
Feulgen	–	–	–	–	–	–
Methyl Green Pyronin	–	–	–	–	–	–

## 4. Discussion

The histochemistry results indicate the inclusion bodies are not composed of nucleic acid or carbohydrate, leaving protein as the most likely major chemical constituent. Immunohistochemistry experiments for detecting Hsc70 showed that the intranuclear inclusions in the renal tubular cells in the immunodeficient mice were typically associated with heat shock cognate 70. Heat shock cognate 71 kDa protein (also known as Hsc70, Hsp73 and heat shock 70 kDa protein 8) is a molecular chaperone that protects cells from the damage caused by physical and chemical hazards [11]. Hsc70 is normally found predominantly within the cellular cytoplasm, but may be recruited to the nuclear compartment following non-specific insults to the nucleus [12]. In complex with a range of other cellular proteins, Hsc70 chaperones the repair process within damaged nuclei [12]. If the nuclear damage can be ameliorated, Hsc70 will exit the nucleus and re-enter the cytoplasm [12]. The detection of Hsc70 within nuclei affected by the murine renal tubular intranuclear inclusions indicates that affected nuclei have been damaged, but does not implicate any particular cause of the insult. The presence of chromatin margination observed by light and electron microscopy also supports the hypothesis that affected cells have incurred some insult, as it is recognised as an early change occurring in the nucleus following irreversible injury, which ultimately leads to cell death [13]. While anti-Hsc70 antibodies appeared to bind to the intranuclear inclusion bodies themselves, it seems that Hsc70 is not the sole constituent of these inclusion bodies. 

Immunohistochemistry experiments using three different antibody preparations for detecting papillomavirus L1 protein revealed that no immunostaining was demonstrable using Camvir, which recognises the seven amino acid long linear epitope GFGAMDF of human papillomavirus (HPV) type 16 L1. Conversely, both polyclonal and monoclonal antibodies for bovine papillomavirus (BPV) type 1 L1 showed strong, specific immunostaining of the intranuclear inclusion bodies. Molecular tests and ultrastructural investigations, in this report and in previous studies, have consistently failed to demonstrate the presence of any virus within affected kidney tissue [2]. The immunohistochemistry results using anti-BPV1 L1 antibodies provide evidence that there is an epitope, antigenically similar enough to BPV1 L1 to permit antibody binding, present within the intranuclear inclusion bodies. 

The monoclonal anti-BPV-1 L1/1H8 antibody found in the mixture with Camvir (Abcam) has not yet had its binding specificity determined. Therefore, it cannot be concluded with any certainty as to which linear or conformational epitope this antibody has bound within the intranuclear inclusion body. The binding specificities of the polyclonal against BPV1 L1 have been described [14], however this product is no longer commercially available.

There is some published evidence of extra-epithelial localization of human papillomaviruses. Bodaghi *et al.* (2005) [15] provided some experimental evidence to support their contention that human papillomaviruses (HPV) may be spread through blood. Subsequently, Chen *et al.* (2009) [16] reported successfully detecting HPV (human papillomavirus) DNA using PCR on peripheral blood of Australian male blood donors, although they suggested the human papillomaviruses they detected were most likely attached to the surface of blood cells, rather than productively infecting the blood cells. 

More controversially, Roperto *et al.* (2012) [17] claimed to have detected productive BPV2 (bovine papillomavirus) infection within the placentas of cows with urinary bladder tumours and in an earlier work, Roperto *et al.* (2011) [18] claimed there was productive BPV2 (bovine papillomavirus) infection of peripheral blood lymphocytes in a cow with papillary carcinoma of the urinary bladder. Based on these previous reports, it is conceivable that a papillomavirus or related virus may indeed be involved in the pathogenesis of murine renal tubular intranuclear inclusions, although this should be considered an unlikely possibility at this stage. 

Rodent species with described papillomaviruses include *Erethizon dorsatum* [3], *Mastomys coucha* [19], *Mesocricetus auratus* [20,21], *Micromys minutus* [22,23], *Mastomys natalensis* [24], *Mus musculus* [20,25,26,27] It remains unlikely that the inclusions were caused by a murine papillomavirus as no other evidence of a murine papillomavirus infection was found.

An alternate hypothesis is that an unknown endogenous murine protein, when concentrated within the nucleus, provides linear or conformational epitopes that antigenically resemble part of the BPV1 L1 protein closely enough, that the anti-BPV1 L1 antibodies used in these experiments cross-react to produce strong, specific positive immunostaining.

Previous reports have indicated that the inclusions have been noted in immunodeficient mice [2,1,28,29]. Here, the six mice were all severely immunodeficient (CBySmn.CB17-*Prkdc^scid^*/J, NOD.CB17-*Prkdc^scid^*/J and B6.129S7-*Rag1^tm1Mom^*/J), but the immunodeficiencies of the CBySmn.CB17-*Prkdc^scid^*/J and B6.129S7-*Rag1^tm1Mom^*/J mice have been produced through different genetic lesions. This indicates that the propensity to develop murine renal tubular intranuclear inclusion bodies appears to be associated with severe immunodeficiency brought about by impaired adaptive immune system function, and is not a direct product of one particular genetic lesion. It is interesting to note that in the present study 5/6 mice were female and 7/8 mice described by Baze and coworkers [2], were also female. The reasons for this apparent sex bias are unclear.

Known nephrotoxins include heavy metals, ochratoxin A [30], aminoglycoside antibiotics, hydrocarbons, non-steroidal anti-inflammatory drugs, some anesthetic agents, oxalates and radiographic contrast media [31]. In our study, it seems unlikely that these agents were involved in the pathogenesis of the inclusion bodies, as the mice had no known exposure to them, the mice were kept in specific pathogen free conditions and most of these agents are not known for producing intranuclear inclusion bodies. Lead exposure, on the other hand, is known to produce intranuclear and intracytoplasmic inclusions in the proximal convoluted tubules of many animal species [32], however, the ultrastructural investigations performed in this study showed that affected nuclei were filled with an electron lucent material, and this is inconsistent with lead inclusions that have an electron dense central core surrounded by a zone of fibrillar structures. Nonetheless, the possibility remains that an unknown environmental insult may have been associated with the formation of renal tubular intranuclear inclusions in these mice.

## 5. Conclusions

In the absence of a more definitive understanding of their composition and pathogenesis, these perplexing inclusions should, for the time being, be considered part of the normal background pathology of immunodeficient mice. Regardless of the precise identity of the binding site for the immunohistochemical reaction, an immunohistochemical method now exists that can be used to confirm and evaluate the histological severity and distribution of suspected cases of murine inclusion body nephropathy. 
